# How Can Immune Checkpoint Inhibitors Cause Hyperprogression in Solid Tumors?

**DOI:** 10.3389/fimmu.2020.00492

**Published:** 2020-03-20

**Authors:** Morgane Denis, Michael Duruisseaux, Marie Brevet, Charles Dumontet

**Affiliations:** ^1^INSERM 1052/CNRS 5286/UCBL - Cancer Research Center of Lyon, Anticancer Antibodies Laboratory, Lyon, France; ^2^Antineo, Lyon, France; ^3^Respiratory Department, Louis Pradel Hospital, Hospices Civils de Lyon Cancer Institute, Bron, France; ^4^Institut de Pathologie Multisites des HCL - Site Est- Hospices Civils of Lyon, Lyon, France

**Keywords:** hyperprogressive disease, immune checkpoint inhibitors, tumor growth, predictive factors, solid tumor

## Abstract

Following the administration of immune checkpoint inhibitors, an unexpected pattern of response designated as hyperprogression may be observed in certain patients. This paradoxical response corresponds to an acceleration in tumor growth and a dramatic decrease of patient survival. The reported incidence rates of hyperprogressive disease are highly variable, ranging between 4 and 29%. In this review, we have performed a literature search on hyperprogressive disease, including both retrospective studies and case reports, and discuss potential predictive biomarkers as well as potential mechanisms associated with immune-checkpoint inhibitor associated hyperprogression.

## Introduction

Since approval by the Food and Drug Administration (FDA) in 2011 of the first antibody, ipilimumab, targeting an immune checkpoint inhibitors (ICI) (1), this class of inhibitors has rapidly developed to include a large variety of cancer indications. Currently approved agents contribute to the activation of anti-tumor cytotoxic T cells by abrogating the immune checkpoint signaling triggered by tumor cells or microenvironment. Monoclonal antibodies targeting CTLA-4 (ipilimumab), PD-1 (nivolumab, pembrolizumab), and PD-L1 (atezolizumab, avelumab, and durvalumab) are currently approved for the treatment of numerous cancers, however, significant responses to immunotherapy remain restricted to a minority of patients and certain tumor types. Unsuccessful treatment may be due to primary resistance or acquired resistance (2–5). In some cases, the disease develops faster than expected and in a more aggressive manner after immune checkpoint targeting immunotherapy. This phenomenon, designated as hyperprogressive disease (HPD), corresponds to a paradoxical boost in tumor growth under treatment and has been described in non-squamous non-small cell lung cancer (NSCLC), head and neck squamous cell carcinoma (HNSCC), urothelial bladder carcinoma, hepatocellular carcinoma, gastric cancer, and anorectal melanoma (6–14), with a rate ranging between 4 and 29%. There are currently few data explaining the occurrence of HPD or allowing clinicians to identify patients at risk of developing HPD. The aim of this review is to provide an update about HPD and potential mechanisms explaining how ICI can induce this phenomenon.

## Immunotherapy Prescription and Monitoring

Immunotherapies targeting immune checkpoints are increasingly used in the relapse setting and are rapidly becoming a component of first-line therapies for melanoma, NSCLC, small cell lung cancer, advanced renal cell carcinoma, triple negative breast cancer, and Merkel cell carcinoma, sometimes in combination with chemotherapy and second line therapies for many tumor types (unresectable and metastatic melanoma, NSCLC, renal cell carcinoma, HNSCC, urothelial carcinoma, colorectal cancer, prostate cancer, and Hodgkin's lymphoma) with durable clinical benefits (15–25). This innovative approach is associated with immune-related adverse events (IRAE) which can be severe (grade 3 or 4) and involve a variety of tissues and organs (26, 27). Early detection of these IRAE as well as appropriate preventive and/or curative therapies have been a major preoccupation for clinicians administering ICIs to their patients.

Unfortunately, only 15–40% of patients benefit from ICIs although some patients will experience long-lasting responses. Melanoma is the only cancer type with a high response rate to single agent ICI therapy (around 40%). A majority of patients display innate resistance to ICI treatment. This may be due to a tumor intrinsic factor, such as loss of HLA expression, target antigen down-regulation or mutation of JAK1/2. Alternatively resistance may be due to extrinsic factors, including pro-tumoral cells such as Treg or myeloid derived suppressor cells present in the tumor microenvironment, or the upregulation of alternative immune checkpoints by effector T cells present in the tumor (2–4, 28).

Accordingly, a major challenge in the field of ICI is the identification of patients which have the greatest chance to benefit from these costly and potentially toxic therapies. A variety of biomarkers have been explored to determine which patients are most likely to respond to therapy (29, 30). PDL1 over-expression has been used as a criterion to prescribe ICIs in patients with NSCLC (18). Additional biomarkers are also considered to predict the responsiveness to treatments, such as FOXP1 methylation status in NSCLC patients or tumor mutation burden (TMB) (31, 32).

Conventional RECIST 1.1 criteria are not optimal to evaluate immunotherapy efficacy (26). Studies have demonstrated that RECIST1.1 criteria under-evaluated the response rate in a series of 160 patients with NSCLC treated by ICIs (33). In a cohort of 655 melanoma patients, RECIST criteria underestimated the benefit of pembrolizumab in 15% of patients (34). This is due in part to the fact that some patients will present pseudoprogression (cf. infra) and will not be considered as responders, at least in the early phases of evaluation of response. Importantly, conventional criteria are not adapted to distinguish patients with pseudoprogression from non-responders, while both categories require distinct patient management. New criteria, created especially for these treatments, such as irRC (immune related Response Criteria) or iRECIST are better adapted to evaluate response to immunotherapy and to discriminate the pseudoprogression profile (26, 35).

## Pseudoprogression

An apparent increase of tumor volume or tumor-associated symptoms has been described in various settings. “Tumor flare” has been described in hormonal therapy of metastatic breast carcinoma and does not require treatment interruption (36). In the case of cytotoxic agents, the concept of pseudoprogression was first described by Brandsma in brain tumors treated by temozolomide, and was defined as an increase of contrast-enhancement and/or edema on MRI without true tumor progression (37). While rarely observed with conventional cytotoxic agents, pseudoprogression is relatively frequent after ICI administration. For tumor types for which there is the largest follow-up after ICI-based therapies, namely melanoma and NSCLC, the pseudoprogressor rates are 3.7–15.8 and 5%, respectively (38–41). This observation was highlighted in advanced NSCLC, Ferrara's study suggesting that pseudoprogression associated with immunotherapies involves a specific mechanism since no pseudoprogression case was observed in the chemotherapy cohort study (10).

Pseudoprogression is totally different from hyperprogression in terms of patient outcome. In the case of ICI therapy, pseudoprogression is defined as an initial increase of tumor size followed by a response to treatment, resulting from an exacerbated immune cell infiltration in the tumor bed, including CD103+ CD8+ cells (42). Pseudoprogression remains a rare response pattern as its average occurrence rate is only 10% in melanoma (11, 41, 43–45). Interestingly, Cohen et al. reported a case of brain metastasis pseudoprogession after pembrolizumab in a patient with melanoma, the histological evaluation having shown that lesions were not true progression but an inflammatory reaction. They identified isolated clusters of tumor cells surrounded by reactive astrocytosis and inflammatory cells (43).

Pseudoprogression has been associated with a high likelihood of 1 year survival when compared to authentic disease progression and partial response patterns, in 21 patients (46). Considering their favorable prognosis, it is important to identify these patients to avoid premature treatment interruption. Unfortunately, detecting pseudoprogressors from non-responders is challenging, and requires additional confirmation by imaging. To address this problem, iRECIST evaluation includes tumor size checkup 4 weeks after disease progression detection, in order to differentiate authentic progression from a pseudoprogression (26, 35).

## Hyperprogression

Hyperprogression, or hyperprogressive disease (HPD), is defined as an accelerated tumor growth after ICI with an increase in the absolute mass of tumor cells superior to what is expected in the setting of conventional progression on treatment, as opposed to pseudoprogression. One of the first publications describing this process reported a patient cohort of 131 patients with various types of cancers from the Gustave Roussy cancer center, 12 of which (9%) were considered as hyperprogressors. In this series HPD was not associated with increased tumor burden at baseline nor with a specific tumor type but was more common in patients older than 65 (6). HPD was defined in this study as a two-fold increase in tumor growth rate after initiation of ICI therapy. Hyperprogression after ICI therapy has been described in multiple type of cancers, including lung, head and neck, anorectal, gastric, and hepatic tumors. Hyperprogression is thus not associated with a single type of cancer (6–14) (Table 1). Most of these patients were diagnosed using RECIST criteria, with the biases involved with this method. HPD was thus observed in 4–29% of cases suggesting that the rate of HPD depends on the type of cancer and is specific to each type of disease.

**Table 1:** Studies reporting hyperprogressive disease patterns.

**References**	**Cancer types**	**Immunotherapy**	**Previous therapies**	**Detection HPD**							**Rate HPD**	**Characteristic/explanation**
				**RECIST1.1**	**TGR**	**TGK**	**TTF**	**Survival HPD**	**Tumor burden**	**Other**		
Champiat et al. (6)	Multiple type of cancer (Melanoma, lung, renal, colorectal, urothelial, lymphoma, HCC)	PD-1/PD-L1 inhibitors	Chemotherapy/radiotherapy/targeted therapy/immunotherapy	Yes	Yes			Median OS 4.6 months (*p =* 0.19)			9% (12/131)	• Older age (*p =* 0.007)
Kato et al. (47)	Multiple type of cancer (Melanoma, NSCLC, SCCHN, CSCC, renal, colorectal)	PD-1/PD-L1/CTLA-4 inhibitors	Chemotherapy/radiotherapy/targeted therapy/immunotherapy				Yes		Yes	Progression pace > two-fold	4% (6/155)	• MDM2/4 amplification (*p =* 0.007) • EGFR alteration (*p =* 0.005)
Saâda-Bouzid et al. (7)	HNSCC	PD-1/PD-L1 inhibitors	ND	Yes		Yes		PFS 2.5 months (*p =* 0.003) RECIST 1.1 and 2.9 months (*p =* 0.02) irRECIST			29% (10/34)	• Presence of cervical nodes at diagnosis (ns) • Presence of regional recurrence (*p* = 0.008)
Faure et al. (9)	Anorectal malignant melanoma	PD-1 inhibitors	Chemotherapy							PET scanner imaging at baseline and after three cycles	Case report	• Hypothesis with a role of monocytes
Ferrara et al. (10)	NSCLC	PD-1/PD-L1 inhibitors	Chemotherapy/radiotherapy	Yes	Yes			Median OS 3.4 months (*p =* 0.03)		Two metastatic sites before PD1/PDL1	14% (56/406)	• Metastatic sites > 2 (*p =* 0.006)
Boland et al. (8)	Epithelial ovarian cancer	PD-1/PD-L1/CTLA-4/LAG3 inhibitors	ND							Very early treatment discontinuation	33.7% (30/89)	• Liver parenchymal metastases (*p =* 0.001) • Neutrophil to lymphocyte ratio > 4 (*p =* 0.017)
Sasaki et al. (13)	Advanced Gastric cancer	PD-1 inhibitors	Chemotherapy/radiotherapy	Yes		Yes		Median OS 2.3 months (*p* < 0.001)	Yes		21% (13/62)	• Absolute neutrophil count increased (*p =* 0.002) • C-reactive protein increased (*p =* 0.006)
Wong et al. (14)	Hepatocellular carcinoma	PD-1/CTLA-4 inhibitors	Chemotherapy/radiotherapy		Yes						Case report	• Hypothesis that previous radiotherapy treatment contributed to HPD
Costantini et al. (12)	NSCLC	PD-1 inhibitors	Radiotherapy	Yes				OS 1.4 months (*p* < 0.0001)		<3 nivolumab injections	20% (57/292)	• PS > 2 at nivolumab initiation (*p* < 0.0001)
Ji et al. (48)	Malignant tumors of digestive system	PD-1/PD-L1/CTLA-4 inhibitors	ND			Yes			Yes		20% (5/25)	ND

Since then, several studies have described HPD on homogeneous cohorts of patients. Ferrara et al. performed a comparative study of 406 patients receiving anti PD1 or anti PDL1 inhibitors for lung cancer, mainly in the relapse setting (93%). These authors found that HPD is more frequent in the cohort of patients treated with immunotherapy, when compared to chemotherapy (13.8 vs. 5.1%) (10). Gandara et al. analyzed 850 patients receiving docetaxel or atezolizumab for NSCLC and found a similar proportion of “fast progressors” in each arm (~10%), suggesting that hyperprogression may result from a very poor prognosis of patients, rather than being due to immunotherapy *per se*. In this study, the number of patients with > 50% growth within 6 weeks was higher in patients receiving anti-PD-L1 therapy (45%, *n* = 20/44) than in those receiving chemotherapy (29%, *n* = 12/41) (49).

In clinical practice defining an HPD remains extremely arduous since only retrospective studies have described this pattern and the acceleration of tumor growth associated with HPD is usually associated with a degradation of the performance status of the patient and death. Moreover, the definition of hyperprogression is not currently consensual. While most studies use the aforementioned definition of HPD, i.e., a tumor growth rate (TGR) twice greater post-treatment than before, other investigators suggest that the tumor growth kinetics (TGK) corresponding of the difference between pre (or post) baseline and baseline of the sum of the largest diameters of the target lesions per unit of time, or a score including multiple parameters such as time to treatment failure (TTF) or appearance of 2 or more new lesions should be used (50–53).

To sum up, HPD appears to be a distinct response pattern. However, it remains unclear whether it is a consequence of immunotherapy or not. HPD is likely to be caused by different mechanisms depending on the cancer type and the immune microenvironment. Thus, we can hypothesize that the nature of the immune microenvironment prior to therapy may play an important role in the occurrence of the HPD phenotype. It has been reported for example that the immune microenvironment is extremely different between lung and gastric cancers. An important determinant of HPD could therefore be the cancer type and its specific microenvironment.

## Factors Predictive of Hyperprogression

There are currently few available data regarding potential biomarkers predictive of an HPD phenotype. Kato and his team first reported the association between MDM2 family member amplification and EGFR aberrations and HPD in a first series of 155 patients, among which six with MDM2/MDM4 amplification demonstrated an HPD phenotype, while 2 out of 10 with EGFR alterations had an HPD phenotype (47). In a molecular profiling study of 102,878 patients, this same group identified the amplification of MDM2 in 3.5%, with large variations among tumor types (63.6% in liposarcoma and <1% in thyroid carcinoma and adenocarcinoma of colon and rectum). Interestingly most patients with MDM2 amplification had a low Tumor Mutational Burden (54).

Other factors have been suggested to constitute risk factors for HPD such as patient age. Champiat et al. observed a higher incidence of HPD in patients older than 65 in their series (6); Sasaki et al. reported that liver metastases, a good performance status and a large sum of target lesion diameters at baseline were associated with a greater risk of HPD (13). These authors also observed that an early increase of neutrophil counts and C reactive protein after initiation of ICIs was only observed in HPD patients. Currently available data for clinical and biological parameters are limited by the retrospective nature of the studies and/or the limited number of patients with an HPD phenotype. Jensen et al. have recently described a genome-wide sequencing of cell-free plasma DNA and suggest that the computed genome instability number (GIN) could help identify HPD, but this study also included a limited number of patients (55). Further prospective studies are needed to confirm these observations and to identify novel markers of HPD.

## Hyperprogression Mechanisms

Several hypotheses have been advanced to explain the underlying mechanisms of hyperprogression. Since HPD is not only a lack of response but an actual acceleration of tumor growth under ICI therapy, it is likely that HPD results from the convergence of several factors including the characteristics of the tumor cells themselves, the status of the patient's immune system and the patient's current or prior therapeutic history.

In the majority of cases, patients received cytotoxic agents before initiation of immunotherapies. In preclinical models we have shown that conventional chemotherapy can in some cases reduce the antitumor activity of immunotherapy (56). We can hypothesize that following chemotherapy treatments, resistant clones were selected due to their ability to escape. It is therefore possible that chemotherapy -resistant clones which are undetectable by immune system are unleashed when ICIs are administrated instead. A better understanding of the possible antagonistic effects of conventional agents and immunotherapy will held apprehend this phenomenon. The lack of currently validated immunomonitoring tools does not allow a predictive evaluation of the patient's pretherapeutic status on the risk of developing HPD.

Alterations in tumor cells induced by ICIs may be involved in the HPD phenotype. We can hypothesize that PD-L1 binding in itself may in some cases cause tumor cell alterations leading to increased progression. Alternatively, some molecular characteristics of tumor cells may be associated with HPD. While JAK1/2 mutations have been shown to be associated with primary resistance to ICIs (4), it is possible that a particular mutation causes HPD, as suggested by Kato with the amplification of MDM2 or the EGFR mutation (47). Xiong et al. analyzed post-therapy HPD tumors and identified somatic mutations in various tumor suppressor genes such as TSC2 and VHL as well as upregulation of oncogenic pathways and reduced immunogenicity (57). An alternative intriguing hypothesis could be that the binding of PDL1 expressed by tumor cells could in itself in some cases enhance tumor cell proliferation.

The role of the immune system, both inside and outside the tumor microenvironment, as a mechanism of hyperprogression remains largely unexplained. Lead suspects are immune cells which favor tumor evasion and progression. Lo Russo et al. analyzed the immune infiltrate of HPD cases and analyzed the evolution of PDX from these patients reimplanted in mice then treated with nivolumab (53). These authors found that pretreatment lesions from all patients classified as HPD showed tumor infiltration by clustered epithelioid macrophages characterized by a CD163+CD33+PD-L1+ profile. Wang et al. showed that tumor-derived exosomes induce PD1+ macrophages which produce IL-10 and block CD8+T cells function (58). Xiong et al. found that innate lymphoid cells 3 (ILC3) are specifically upregulated in HPD tumors (57). Innate lymphoid cells respond to cytokine stimulation in the absence of a specific antigen. Dual roles are described for this particular cell type. ILC3 have been reported to secrete IL-17, IL-22, and GM-CSF, and thus can support cancer development (59). In a colon cancer mouse model it has been shown that depletion of IL-22 produced by ILC3 cells reduced the growth of gastro-intestinal cancers (60). Irshad et al. reported a correlation between the presence of ILC3 cells in the tumor microenvironment and an increased risk of lymph node metastasis in breast cancer (61). Conversely Carrega et al. reported that natural cytotoxicity receptors were present in ILC3 cells and that these NCR+ILC3 cells contributed to the formation of tertiary lymphoid structures (TLS) which were associated with less advanced tumor stages in NSCLC patients (62). Therefore, the potential role of ICL3 in hyperprogression needs to be analyzed in depth.

Zuazo-Ibarra et al. analyzed circulating “highly differentiated human cells” (T_HD_) defined by a CD28-CD27-CD4+ phenotype, both at baseline and after therapy. They concluded that low baseline T_HD_ values identified non-responders and HPD patients, with a proliferative burst of this cell type under therapy (63). Kamada et al. analyzed the role of PD-1 regulatory T cells in gastric cancer patients and found that HPD patients underwent a marked increase in intratumoral proliferating Tregs (64). It is possible to imagine that if PD-1 regulatory T cells are present; these may be activated by ICI therapy. PD-1 regulatory T cells can proliferate and inhibit anti-tumor immune cells. This vicious circle would then allow an exacerbated tumor progression. This mechanism is called contra-suppression in immunoregulation (65, 66). This may suppose that other pro-tumoral cells are upregulated and amplify this phenomenon.

## Conclusion

**Figure 1:**
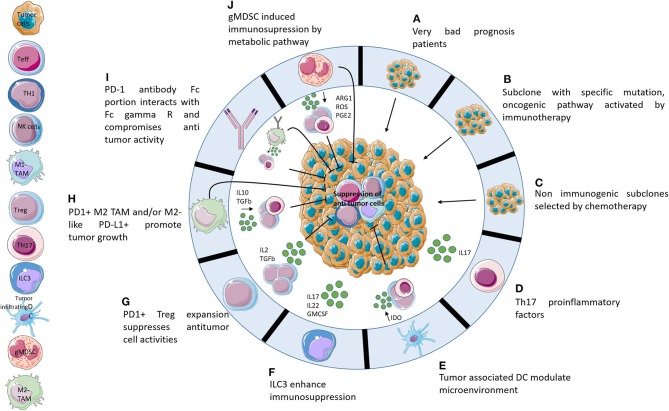
Potential hypotheses explaining hyperprogression. Ten potential mechanisms that may be responsible for hyperprogression following administration of immunotherapies. **(A)** HPD is not caused by immunotherapies but it is a consequence of adverse prognostic profiles. **(B)** Activation of oncogenic pathways caused by PD-1/PD-L1 axis blockade. **(C)** Non-immunogenic subclones resistant to chemotherapy develop very quickly following the cessation of chemotherapy. **(D)** The TH17 axis causes increased inflammation following immunotherapy administration. **(E)** Tumor associated DCs contribute to immunosuppression of the microenvironment after blocking of the PD-1/PD-L1 axis. **(F)** PD1/PD-L1 blockade activates ILC3 which enhances immunosuppression by protumoral interleukins. **(G)** Blocking of PD-1 activates Treg PD1+ which induces suppression of Teff. **(H)** Activation, by PD-1/PD-L1 axis blocking, of M2-like PD-L1+ cells which promote tumor growth directly and indirectly by expansion of protumoral cells. **(I)** Fc receptor of anti-PD-1 enhances tumor growth by recruitment of M2-like cells. **(J)** gMDSC following immunotherapies induces immunosuppression by release metabolites which suppress antitumor cells.

There is a growing consensus that the HPD phenotype is a clinically meaningful entity describing patients whose evolution and prognosis is worse in case of ICI therapy. Additional studies are required to better understand the concept of hyperprogression and hopefully prevent it or identify patients at risk. Many hypotheses remain to be elucidated (Figure 1). To clarify the mechanism, extensive studies of the tumor and immune microenvironment should be performed. A study with pre- and post-treatment patient samples will help to decipher the underlying mechanisms and identify new biomarkers. However, the hyperprogression phenotype being rare, it will take very large cohorts of patients to first identify then validate these observations. Thus, *in vivo* murine models may be a good alternative to generate hypotheses, as some highly resistant syngeneic models mimic hyperprogression. Characterizing the immune and tumor microenvironment of syngeneic tumor mouse models may provide initial explanations of the mechanisms involved (53).

## Author Contributions

MDe wrote the first draft of the manuscript. All authors contributed to manuscript revision, read, and approved the submitted version.

### Conflict of Interest

MDe is employed by the company Antineo. MDu reports personal fees for advisory board participation: Roche, Bristol-Myers Squibb, Takeda, Boehringer Ingelheim, Merck Sharp & Dohme, AstraZeneca, and AbbVie, Pfizer and grants for institutional research project: Nanostring, Blueprint, Boehringer Ingelheim. The remaining authors declare that the research was conducted in the absence of any commercial or financial relationships that could be construed as a potential conflict of interest.
